# Diseases of Women

**Published:** 1889-03

**Authors:** 


*Diseases of Women.* By A. H. N. LEWERS, M.D. Pp. 400. London: H. K. LEWIS. 1888.

Whatever just cause of complaint women may have as to their being overlooked in suffrage, science, art, or matrimony, they certainly cannot complain that they are neglected as to their diseases. Was this book wanted? Does it fill a void, narrow or gaping? While American and German specialists are sending forth large original works, and even encyclopædias, on the diseases of women, English specialists seem to content themselves with pouring forth numerous small works, abstracted, condensed and modified from the originals.

Although we believe that Dr. Lewers would have been doing more for science and his own reputation by thoroughly and exhaustively working out one feature of one disease, than by superficially traversing the whole field, we must admit that this work is, on the whole, well done, and worthily occupies its place in Lewis's Practical Series.

